# Patterns of cardiometabolic burden by age and education and their relation to objective cognition in adults with subjective cognitive complaints

**DOI:** 10.25122/jml-2026-0136

**Published:** 2026-09

**Authors:** Diana Chira, Olivia Verișezan-Roșu, Ștefan Strilciuc, Dafin Fior Mureșanu

**Affiliations:** 1 Department of Neurosciences, Iuliu Hațieganu University of Medicine and Pharmacy, Cluj-Napoca, Romania; 2 RoNeuro Institute for Neurological Research and Diagnostic, Cluj-Napoca, Romania; 3 Research Center for Functional Genomics, Biomedicine and Translational Medicine, Iuliu Hațieganu University of Medicine and Pharmacy, Cluj-Napoca, Romania; 4 Department of Translational Neurosciences, MEDFUTURE, Iuliu Hațieganu University of Medicine and Pharmacy, Cluj-Napoca, Romania

**Keywords:** subjective cognitive complaints, cardiometabolic burden, cognitive reserve, educational attainment, attention, working memory, aging

## Abstract

Cardiometabolic multimorbidity is associated with cognitive decline, but its distribution and relationship with cognitive performance in adults with subjective cognitive complaints are less clear. We analyzed baseline data from 276 adults aged 50–75 years with such complaints and a Montreal Cognitive Assessment score >25 enrolled in the N-HANCE trial. A cardiometabolic burden score (0–3) was derived from self-reported hypertension, any lipid disorder and diabetes. Cognitive performance was assessed using three Test of Attentional Performance subtests and Digit Span Forward and Backward. Poisson regression examined associations of age, sex, and education with the burden score, and multivariable linear regression examined associations of burden with cognition, with Benjamini–Hochberg correction across five outcomes. Hypertension was reported by 52.5% of participants, a lipid disorder by 46.7% and diabetes by 9.1%. Mean burden increased across age groups (0.83, 1.23 and 1.40; *P* < 0.001) and decreased across educational categories (1.33, 1.12 and 0.82 from low to higher education; *P* < 0.001). Both associations remained after adjustment for sex and for one another (age: incidence rate ratio [IRR] = 1.033; 95% CI, 1.016–1.049; education: IRR = 0.888; 95% CI, 0.830–0.949). Burden was not associated with any cognitive outcome after adjustment for age, sex, and education (all Benjamini–Hochberg-adjusted *P* ≥ 0.871), whereas education was associated with all five outcomes (all *P* < 0.001). In this cohort, cardiometabolic burden varied by age and education but showed no detectable cross-sectional association with the cognitive measures used. Longitudinal studies with objective cardiometabolic measurements are needed to examine its relationship with subsequent cognitive change.

## INTRODUCTION

Subjective cognitive complaints are often described as self-perceived difficulties with memory, attention, or other cognitive functions. They are increasingly recognized as a common experience among middle-aged and older adults [1]. In recent years, growing attention has been directed toward these complaints because they may represent one of the earliest manifestations of cognitive decline, even before deficits can be measured on standardized neuropsychological assessments [1–3].

Cardiometabolic disorders, such as hypertension, diabetes mellitus, dyslipidemia, obesity, and cardiovascular disease, are among the most consistently identified modifiable risk factors for cognitive impairment and dementia [4]. These conditions do not act independently. Their combined presence, referred to as cardiometabolic multimorbidity, is recognized as an important factor influencing brain health throughout life. Growing evidence suggests that having multiple vascular and metabolic conditions may negatively affect brain aging and increase the risk of cognitive decline and dementia [5–8].

Several mechanisms may explain the relationship between cardiometabolic disorders and cognitive decline. Prolonged exposure to vascular and metabolic risk factors can lead to reduced blood flow to the brain, chronic inflammation and other changes that adversely affect brain structure and function [9–11]. Consequently, individuals with multiple cardiometabolic conditions tend to have poorer cognitive performance and face a higher risk of brain atrophy, mild cognitive impairment and dementia [10].

Aging is accompanied by a progressive increase in the prevalence of vascular and metabolic disorders, resulting in a greater burden of multimorbidity among older individuals [12,13]. However, factors beyond age alone can contribute to the development and accumulation of cardiometabolic conditions. Educational attainment, often viewed as a marker of cognitive reserve, is an important determinant of healthy aging [14,15]. Individuals with higher levels of education tend to adopt healthier lifestyles, engage more in preventive health behaviors, and have better access to healthcare [16]. These factors may contribute to greater resilience to age-related cognitive changes and influence both cardiometabolic health and the impact of age-related brain pathology [14,17,18].

Despite the recognized importance of both age and educational attainment, little is known about how these factors shape cardiometabolic burden specifically among adults with subjective cognitive complaints, a population in which such burden may be particularly consequential, given that its members already display an early marker of cognitive vulnerability. Furthermore, whether cardiometabolic burden at this pre-impairment stage is already associated with a measurable decrement in objective cognitive performance has not been established.

Cardiometabolic multimorbidity is usually defined as the co-occurrence of established diseases such as diabetes, heart disease and stroke. Here we consider instead the accumulation of self-reported cardiometabolic risk conditions (hypertension, lipid disorders and diabetes), which we refer to as cardiometabolic burden. In a previous analysis of this cohort, we reported that educational attainment and several lifestyle and psychosocial factors were associated with objective performance [19]. Cardiometabolic conditions were not examined in that report. The present study therefore had two aims: to characterize the distribution of cardiometabolic burden according to age and educational attainment in a well-characterized cohort of adults with subjective cognitive complaints, and to examine whether this burden is associated with objective cognitive performance in attention and working memory. We hypothesized that higher cardiometabolic burden would be associated with poorer cognitive performance. Whether educational attainment moderates this association was examined as an exploratory question.

## MATERIAL AND METHODS

### Study design and participants

This study is a cross-sectional analysis of baseline data from the N-HANCE study, a prospective, randomized, double-blind, placebo-controlled trial evaluating N-PEP-12 dietary supplementation in middle-aged and older adults with subjective cognitive complaints (ISRCTN10514154) [20]. All data analyzed were collected at the screening and baseline visit, prior to randomization and before any participant received the study product.

Participants were recruited from the local community and assessed at the RoNeuro Institute for Neurological Research and Diagnostic, Cluj-Napoca, Romania, between March 2023 and June 2025. Eligible participants were aged 50 to 75 years, reported subjective cognitive complaints, and had no clinically significant cognitive impairment, defined as a Montreal Cognitive Assessment (MoCA) score above 25. Further inclusion criteria required a body mass index (BMI) between 18.0 and 29.9 kg/m^2^, general good health confirmed by clinical examination, and sufficient literacy to complete the neuropsychological assessments. Exclusion criteria comprised clinically significant systemic disease, active major neurological disease, alcohol consumption exceeding 14 units weekly, drug abuse within 5 years, bariatric surgery, participation in another trial within three months, and functional impairment liable to interfere with cognitive testing. These criteria have direct consequences for the interpretation of the prevalence estimates reported below and are addressed in the Discussion: the sample is, by construction, healthier than the general Romanian population of comparable age.

Subjective cognitive complaints were ascertained at screening through a face-to-face semi-structured clinical interview conducted by a clinician with each participant, covering memory, attention, executive function, language and visuospatial abilities. No standardized questionnaire was administered, no numerical cut-off was applied, and no minimum duration or persistence of complaints was required. Participants were therefore not selected according to formal research criteria for subjective cognitive decline, and the broader term “subjective cognitive complaints” is used throughout for this reason.

The study was approved by the Ethics Committee of the Iuliu Hațieganu University of Medicine and Pharmacy, Cluj-Napoca (AVZ 294, 28 November 2022) and conducted in accordance with the Declaration of Helsinki. All participants provided written informed consent.

### Cardiometabolic variables and burden score

Cardiometabolic conditions were ascertained from self-reported medical history recorded at the baseline visit, as present or absent. Four conditions were recorded: hypertension, hypercholesterolemia, dyslipidemia and diabetes mellitus.

Hypercholesterolemia and dyslipidemia were captured as separate fields in the electronic case report form. Because hypercholesterolemia is a type of dyslipidemia, counting both could double-count lipid disorders, giving them more weight than hypertension or diabetes on the basis of how the medical history was recorded rather than disease severity. For the primary analysis, the two fields were therefore collapsed into a single binary indicator of any lipid disorder, present if either field was positive.

The cardiometabolic burden score was computed as the simple count of three conditions (hypertension, any lipid disorder and diabetes), yielding a score from 0 to 3. A four-item version treating hypercholesterolemia and dyslipidemia separately was retained for sensitivity analysis. Other cardiovascular conditions were recorded in the medical history (cardiac arrhythmia, *n* = 20; heart failure, *n* = 7; hypotension, *n* = 10) but were not included in the burden score, because the score was intended to capture cardiometabolic risk conditions rather than established cardiac disease, and because their prevalence was low. All participants, including those reporting these conditions, met the eligibility criterion of no clinically significant systemic disease.

### Cognitive assessment

Objective cognitive performance was assessed with two instruments. The Test of Attentional Performance (TAP) [21] was administered in three subtests: Alertness (median reaction time without a warning signal), Divided Attention (omissions), and Working Memory (omissions). Results are expressed as normed T-scores, scaled so that higher values indicate better performance. Values falling below the instrument floor of 20 were recoded to 19 prior to analysis, preserving the ordinal ranking of below-floor performance without introducing implausible values. Because TAP T-scores are normed according to age, sex, and educational level, these demographic characteristics are incorporated into the derivation of the standardized scores. We nevertheless retained age, sex and educational attainment as covariates in the regression models to account for potential residual differences between the study sample and the normative population.

Furthermore, Digit Span Forward and Backward from the Wechsler Adult Intelligence Scale, Fourth Edition [22], were administered and scored as raw totals. These scores are not normed and were therefore analyzed with age, sex and education as covariates.

### Sociodemographic variables

Age was recorded in years and additionally categorized into three groups (50–59, 60–69 and 70–75 years). Educational attainment was coded according to the International Standard Classification of Education (ISCED). Levels were recorded on a 1–9 scale corresponding to ISCED 0–8; observed values ranged from 2 to 9 (ISCED 1 to ISCED 8). The variable was retained as ordinal for regression and correlation analyses, with coefficients expressed per ISCED level. For descriptive comparison, it was collapsed into three categories: low (ISCED 1–3, up to upper secondary education), intermediate (ISCED 4–5, post-secondary non-tertiary and short-cycle tertiary education), and higher (ISCED 6–8, bachelor’s level and above). Sex and BMI were also recorded.

### Statistical analysis

Analyses and visualizations were performed in SAS OnDemand for Academics (version 9.4). Continuous variables are summarized as mean ± standard deviation (SD), and categorical variables as counts and percentages. Associations between the burden score and ordinal or continuous variables were assessed by Spearman rank correlation. Comparisons of the burden score across age and education groups used the Kruskal–Wallis test. The prevalence of individual conditions across groups was compared by Pearson’s chi-square test.

Because age and educational attainment are themselves correlated and each may confound the apparent effect of the other, the joint correlates of cardiometabolic burden were examined in a Poisson regression of the burden count on age, education and sex, with results expressed as incidence rate ratios. Model adequacy was assessed by the ratio of deviance to degrees of freedom.

The association between cardiometabolic burden and objective cognitive performance was examined by multivariable linear regression, with each cognitive outcome regressed on the burden score with age, sex and educational attainment as covariates. Across the five cognitive outcomes, *P* values were corrected for multiple comparisons using the Benjamini–Hochberg false discovery rate procedure. To allow direct comparison of effect magnitudes across outcomes measured on different scales, standardized coefficients (change in the outcome, in SD units, per 1-SD increase in the predictor) were additionally computed for educational attainment and for cardiometabolic burden and are presented graphically.

As an exploratory question, we examined whether educational attainment, as a proxy for cognitive reserve, might moderate the relationship between cardiometabolic burden and cognitive performance. This was tested by adding a burden × education interaction term to each model. Because the study was not designed to detect interaction effects and is underpowered for effects of small to moderate magnitude, these analyses are reported as exploratory.

Sensitivity analyses repeated the principal models using the four-item burden score. There were no missing data, and all analyses included the full sample of 276 participants. All tests were two-tailed, with significance set at *P* < 0.05.

## RESULTS

### Sample characteristics

The analysis included all 276 participants enrolled in the N-HANCE study. Mean age was 61.4 ± 7.4 years (range, 50–75 years), and 199 participants (72.1%) were women. Mean BMI was 27.00 ± 2.27 kg/m^2^. Educational attainment was distributed as follows: higher education, 119 (43.1%); intermediate education, 34 (12.3%); and low education, 123 (44.6%). Educational distribution did not differ across age groups (*P* = 0.803; Table 1).

**Table 1:** Sociodemographic and clinical characteristics of the study population, overall and by age group

Variable	Total (*N* = 276)	50–59 years (*n* = 121)	60–69 years (*n* = 112)	70–75 years (*n* = 43)	*P*
Age, years, mean ± SD	61.4 ± 7.4	54.1 ± 3.0	65.2 ± 2.7	72.2 ± 1.8	—
Female, *n* (%)	199 (72.1)	92 (76.0)	80 (71.4)	27 (62.8)	0.246
BMI, kg/m^2^, mean ± SD	27.00 ± 2.27	26.63 ± 2.42	27.39 ± 2.08	27.00 ± 2.23	**0.013**
Educational attainment, *n* (%)					0.803
Higher	119 (43.1)	55 (45.5)	45 (40.2)	19 (44.2)	
Intermediate	34 (12.3)	13 (10.7)	14 (12.5)	7 (16.3)	
Low	123 (44.6)	53 (43.8)	53 (47.3)	17 (39.5)	

Data are presented as mean ± SD or *n* (%). *P* values are from the chi-square test (categorical variables) or the Kruskal–Wallis test (continuous variables). Bold *P* values indicate statistical significance (*P* < 0.05). BMI, body mass index; SD, standard deviation.

### Prevalence of cardiometabolic conditions

Hypertension was the most frequently reported condition, present in 145 participants (52.5%). Hypercholesterolemia was reported by 121 (43.8%), and dyslipidemia by 70 (25.4%); taken together, 129 participants (46.7%) reported at least one lipid disorder. Diabetes mellitus was reported by 25 participants (9.1%; Table 2).

**Table 2:** Prevalence of cardiometabolic conditions and burden score by age group and educational attainment

Condition	Total (*N* = 276)	50–59 years (*n* = 121)	60–69 years (*n* = 112)	70–75 years (*n* = 43)	*P*	Higher education (*n* = 119)	Intermediate education (*n* = 34)	Low education (*n* = 123)	*P*
Hypertension, *n* (%)	145 (52.5)	43 (35.5)	69 (61.6)	33 (76.7)	**< 0.001**	47 (39.5)	21 (61.8)	77 (62.6)	**0.001**
Hypercholesterolemia, *n* (%)	121 (43.8)	49 (40.5)	53 (47.3)	19 (44.2)	0.576	43 (36.1)	14 (41.2)	64 (52.0)	**0.042**
Dyslipidemia, *n* (%)	70 (25.4)	22 (18.2)	32 (28.6)	16 (37.2)	**0.029**	24 (20.2)	8 (23.5)	38 (30.9)	0.154
Any lipid disorder, *n* (%)	129 (46.7)	51 (42.1)	56 (50.0)	22 (51.2)	0.398	44 (37.0)	15 (44.1)	70 (56.9)	**0.008**
Diabetes mellitus, *n* (%)	25 (9.1)	7 (5.8)	13 (11.6)	5 (11.6)	0.246	7 (5.9)	2 (5.9)	16 (13.0)	0.122
Burden score, mean ± SD	1.08 ± 0.91	0.83 ± 0.87	1.23 ± 0.89	1.40 ± 0.90	**< 0.001**	0.82 ± 0.86	1.12 ± 0.84	1.33 ± 0.91	**< 0.001**

The burden score is the count of hypertension, any lipid disorder, and diabetes (range 0–3). *P* values are from the chi-square test (conditions) or the Kruskal–Wallis test (burden score). Bold *P* values indicate statistical significance (*P* < 0.05). SD, standard deviation.

Prevalence across age groups differed markedly by condition. Hypertension showed a steep age gradient, rising from 35.5% at 50–59 years to 61.6% at 60–69 years and 76.7% at 70–75 years (*P* < 0.001). Dyslipidemia also increased with age (18.2%, 28.6% and 37.2%; *P* = 0.029), whereas any lipid disorder (*P* = 0.398) and diabetes (*P* = 0.246) did not vary significantly.

Across educational categories, hypertension (39.5%, 61.8% and 62.6% from higher to low education; *P* = 0.001), hypercholesterolemia (*P* = 0.042) and any lipid disorder (37.0%, 44.1% and 56.9%; *P* = 0.008) were all significantly more frequent in participants with lower educational attainment. Diabetes showed the same pattern without reaching statistical significance (5.9%, 5.9% and 13.0%; *P* = 0.122).

### Distribution of cardiometabolic burden

The three-item burden score was distributed as follows: 90 participants (32.6%) had no cardiometabolic condition, 86 (31.2%) had one, 87 (31.5%) had two, and 13 (4.7%) had all three. Mean burden was 1.08 ± 0.91 (Figure 1AB).

**Figure 1:**
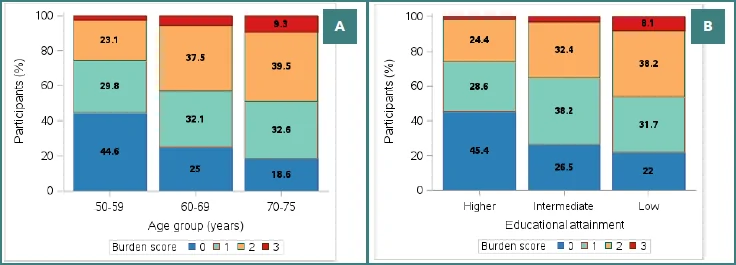
Distribution of the cardiometabolic burden score by age group and educational attainment. (A) Age group; (B) educational attainment. Values within bars are percentages of participants within each group.

Burden increased significantly across age groups (Kruskal–Wallis *H* = 17.18; *P* < 0.001; Spearman ρ = 0.308 with age in years; *P* < 0.001) and decreased significantly across educational categories (*H* = 18.09; *P* < 0.001; Spearman ρ = −0.281 with ISCED level; *P* < 0.001).

Age and educational attainment were only weakly correlated with one another (ρ = −0.122; *P* = 0.043), indicating that the two gradients are not simply reflections of a single underlying variable.

### Age and education as independent correlates of cardiometabolic burden

In a Poisson regression of burden count on age, educational attainment and sex, both age and education remained independently associated with burden. Each additional year of age was associated with a 3.3% higher burden count, and each additional ISCED level with an 11.2% lower burden count. Sex was not associated with burden (*P* = 0.988; Table 3).

**Table 3:** Poisson regression of cardiometabolic burden score on age, educational attainment and sex

Predictor	IRR	95% CI	*P*
Age (per year)	1.033	1.016–1.049	**< 0.001**
Education (per ISCED level)	0.888	0.830–0.949	**< 0.001**
Male (vs female)	1.002	0.777–1.292	0.988

Estimates are from a Poisson regression model. Bold *P* values indicate statistical significance (*P* < 0.05). CI, confidence interval; IRR, incidence rate ratio; ISCED, International Standard Classification of Education.

The persistence of both effects after mutual adjustment indicates that the educational gradient in cardiometabolic burden is not attributable to confounding of education by age, and vice versa. The Poisson model showed no evidence of overdispersion (deviance/*df* = 0.91; Pearson χ^2^/*df* = 0.72). The model-based standard errors are, if anything, conservative, because the dispersion statistics below 1 show modest underdispersion, as would be expected for a count bounded at three.

### Cardiometabolic burden and objective cognitive performance

In unadjusted analyses, cardiometabolic burden showed weak negative correlations with Digit Span Backward (ρ = −0.175; *P* = 0.004), Digit Span Forward (ρ = −0.144; *P* = 0.017) and the TAP Working Memory T-score (ρ = −0.119; *P* = 0.047). Correlations with TAP Alertness and TAP Divided Attention were not statistically significant. Negative coefficients indicate poorer performance with higher burden.

These associations did not persist after adjustment. In multivariable linear regression with age, sex and educational attainment as covariates, cardiometabolic burden was not associated with any cognitive outcome, and no result approached significance after Benjamini–Hochberg correction (Table 4). The confidence intervals were centered near zero across all five outcomes. Expressed in SD units of each outcome, they excluded associations larger than approximately 0.2 SD per additional cardiometabolic condition in either direction, indicating that a large association per condition is unlikely. A small association, which could accumulate to a moderate difference between participants with no condition and those with three, cannot be excluded.

**Table 4:** Cardiometabolic burden and objective cognitive performance

Outcome	Mean ± SD	Unadjusted ρ	*P*	Adjusted β	95% CI	*P*	BH-adjusted *P*
TAP Alertness (T)	36.96 ± 8.70	−0.097	0.110	0.029	−1.151 to 1.209	0.961	0.961
TAP Divided Attention omissions (T)	41.53 ± 10.31	−0.057	0.348	0.327	−1.068 to 1.722	0.645	0.871
TAP Working Memory omissions (T)	42.84 ± 13.95	−0.119	**0.047**	−0.818	−2.715 to 1.079	0.397	0.871
Digit Span Forward	7.54 ± 2.11	−0.144	**0.017**	−0.055	−0.333 to 0.223	0.697	0.871
Digit Span Backward	5.40 ± 1.83	−0.175	**0.004**	−0.062	−0.291 to 0.167	0.593	0.871

Higher T-scores indicate better performance. β expressed per additional cardiometabolic condition, adjusted for age, sex, and educational attainment; Bold *P* values indicate statistical significance (*P* < 0.05). BH, Benjamini–Hochberg; CI, confidence interval; SD, standard deviation; T, T-score; TAP, Test of Attentional Performance.

### Educational attainment and cognitive performance

By contrast, and consistent with our previous report in this cohort [19], educational attainment was strongly associated with performance on every cognitive measure after adjustment for age and sex (Table 5; Figure 2).

**Figure 2:**
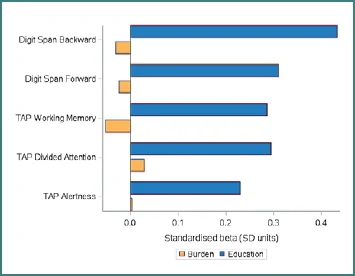
Standardized regression coefficients for educational attainment and for cardiometabolic burden across the five cognitive outcomes, on a common scale. Education was associated with cognitive performance; cardiometabolic burden was not. SD, standard deviation; TAP, Test of Attentional Performance

**Table 5:** Educational attainment and objective cognitive performance, adjusted for age and sex

Outcome	β per ISCED level	95% CI	*P*
TAP Alertness (T)	1.115	0.560–1.671	**< 0.001**
TAP Divided Attention omissions (T)	1.689	1.031–2.346	**< 0.001**
TAP Working Memory omissions (T)	2.226	1.331–3.121	**< 0.001**
Digit Span Forward	0.365	0.234–0.496	**< 0.001**
Digit Span Backward	0.441	0.334–0.549	**< 0.001**

β is the change in the outcome per ISCED level. Bold *P* values indicate statistical significance (*P* < 0.05). CI, confidence interval; ISCED, International Standard Classification of Education; T, T-score; TAP, Test of Attentional Performance.

These associations are notable for the TAP measures in particular, because TAP T-scores are already normed for educational level and a residual association with education was therefore not expected. Because its magnitude depends on the normative adjustments applied to these scores, the finding should be interpreted cautiously and is considered further in the Discussion.

### Exploratory analysis: education as a moderator

The burden × education interaction was not statistically significant for Digit Span Forward (*P* = 0.492), Digit Span Backward (*P* = 0.718), TAP Divided Attention (*P* = 0.347) or TAP Working Memory (*P* = 0.939). A nominally significant interaction was observed for TAP Alertness (uncorrected *P* = 0.011; *P* = 0.057 after Benjamini–Hochberg correction across the five interaction tests). Its direction, however, was opposite to that predicted by the cognitive reserve hypothesis: the interaction coefficient was negative (β = −0.790), indicating that any apparent advantage associated with cardiometabolic burden diminished as educational attainment increased, rather than educational attainment buffering an adverse effect of burden. Given this direction, the multiplicity of testing, the education-normed nature of the outcome and the limited power for interaction effects, the finding should be interpreted cautiously and does not provide evidence supporting the hypothesized moderating effect.

### Sensitivity analyses

Repeating the principal cognitive models with a four-item burden score, treating hypercholesterolemia and dyslipidemia separately, yielded the same conclusions (all *P* ≥ 0.256).

## DISCUSSION

This cross-sectional analysis of 276 middle-aged and older adults with subjective cognitive complaints examined the distribution of cardiometabolic burden and its relationship with objective cognitive performance. Two main findings emerge. Cardiometabolic burden increased with age and decreased with educational attainment, and both gradients persisted after mutual adjustment. Cardiometabolic burden, however, showed no association with any cognitive outcome once age, sex, and education were accounted for. Educational attainment, by contrast, was associated with performance on every cognitive measure, in line with our previous report in this cohort [19].

### Cardiometabolic burden was not associated with cognitive performance

We hypothesized that a higher cardiometabolic burden would be associated with poorer objective cognitive performance in this population. This hypothesis was not supported by the adjusted analyses.

The results are nevertheless informative. In the unadjusted analyses, cardiometabolic burden was weakly but significantly associated with both Digit Span measures and with working memory omissions. Considered alone, these findings could appear to support our initial hypothesis. However, none of these associations remained significant after adjustment for age, sex, and educational attainment. This suggests that the initial associations were largely explained by the fact that both cardiometabolic burden and cognitive performance were related to age and education in this sample.

The confidence intervals help place the null findings in context, although they cannot rule out all potentially meaningful differences. For example, for Digit Span Backward, the confidence interval ranged from −0.291 to 0.167 points per additional cardiometabolic condition. Therefore, the estimates do not support a large cross-sectional association with Digit Span Backward in this sample. Across outcomes, however, an association of up to about 0.2 SD per condition remains compatible with the data, so small effects of the size reported in larger cohorts cannot be excluded. This finding differs from the well-established association between cardiometabolic multimorbidity and cognitive decline reported in older populations [5,7], as well as from the more recent finding that cardiometabolic multimorbidity is associated with a higher prevalence of subjective cognitive decline [6]. These studies, however, defined multimorbidity by established diseases or examined subjective rather than objective outcomes, and most assessed longitudinal decline, so they are not directly comparable with a cross-sectional count of risk conditions. Several factors may explain this difference.

First, the characteristics of our sample may have limited the severity and variability of cardiometabolic disease. Eligibility required the absence of clinically significant systemic disease and a BMI below 30 kg/m^2^. Participants with cardiometabolic conditions were therefore individuals who met the parent trial’s health-related eligibility criteria, rather than a population selected for advanced or decompensated disease. Most reported conditions were also likely to have been diagnosed and under treatment, since eligibility required general good health confirmed by clinical examination. A treated and reasonably well-controlled condition would be expected to relate less strongly to cognitive performance than an untreated or poorly controlled one, and treatment status was not recorded in the present dataset, as noted in the Limitations. Studies relying on self-reported vascular risk factors have likewise reported heterogeneous and often domain-specific associations with cognition. In the Wisconsin Longitudinal Study, self-reported hypertension showed minimal to no association with cognitive performance after adjustment for other cardiovascular risk factors [23], whereas a remote-assessment study found that hypertension was associated with episodic memory but observed no associations for diabetes or high cholesterol [24]. Our findings therefore address a relatively specific question: whether self-reported cardiometabolic conditions are associated with the cognitive measures used in otherwise relatively healthy adults. In this context, we did not observe such an association.

Second, the duration of cardiometabolic exposure may be relevant. Cardiometabolic risk factors are thought to affect cognition through cumulative vascular and metabolic processes that develop over many years [9–11]. Previous research has also suggested that midlife cardiometabolic risk may be more strongly related to later cognitive outcomes than cardiometabolic status measured at older ages [25,26]. In our study, medical history indicated whether a condition was present, but did not provide detailed information on how long the condition had been present. A participant diagnosed with hypertension two years earlier and one living with hypertension for twenty years would therefore both contribute to the same measure of burden, despite potentially having very different cumulative exposure.

Third, the cognitive eligibility criteria may have restricted the range of cognitive performance and reduced our ability to detect small associations. Participants were required to have a MoCA score above 25, thereby excluding individuals with lower global cognitive performance. Although the computerized tasks provided domain-specific measures of attention and working memory, cardiometabolic effects within a cognitively relatively healthy sample may be subtle. In a substantially larger cohort of 2480 cognitively unimpaired middle-aged adults, a higher number of self-reported cardiovascular risk factors was associated with poorer attention and memory, but the observed associations were small [27]. Other research using computerized cognitive assessment has also reported associations that differed across individual cardiometabolic conditions and cognitive domains [24]. Thus, the restricted cognitive range, sample size, and correction for multiple comparisons in the present study may have limited the detection of small or domain-specific effects.

Fourth, cardiometabolic burden may be related to perceived cognitive change before it becomes detectable on performance testing. Longitudinal follow-up would be required to establish whether measurable differences emerge later [28]. The contrast with the finding of Zhu *et al*. [6], who reported an association between cardiometabolic multimorbidity and subjective cognitive decline, may also be relevant in this regard. Because all participants in the present study reported cognitive complaints, this possibility could not be tested here. This would also be consistent with evidence suggesting that subjective cognitive complaints can be influenced by affective and health-related factors and do not always correspond closely to objective cognitive performance [29].

These interpretations should be considered cautiously. The cross-sectional design, the lack of information on the duration and severity of individual cardiometabolic conditions, and the relatively healthy characteristics of the sample limit our ability to distinguish between these possible explanations.

### Age and education are independently associated with cardiometabolic burden

Cardiometabolic burden increased with age and was lower among participants with higher educational attainment. In the Poisson regression analysis, both associations remained significant after adjustment for the other variables and for sex, suggesting that age and education were independently associated with cardiometabolic burden in this sample.

The increase in burden across age groups was mainly driven by hypertension, which was more than twice as common in the oldest compared with the youngest age group. Dyslipidemia also contributed to the age-related pattern, although to a lesser extent, whereas diabetes did not differ significantly across age groups. This pattern is broadly consistent with the epidemiology of these conditions. Hypertension becomes increasingly common with age, while diabetes is influenced by a wider range of factors, including adiposity and genetic susceptibility [30–32]. The exclusion of participants with obesity by the study eligibility criteria may also explain the lack of a clear age-related pattern for diabetes [12]. For context, Romanian population surveys have reported hypertension in approximately 45% of adults [33] and diabetes in approximately 12% [34]. The present figures (52.5% and 9.1%) are not directly comparable, because diagnoses were self-reported in a screened sample of adults aged 50–75 years.

The association with educational attainment was observed for the overall cardiometabolic burden as well as for several individual conditions. Hypertension, hypercholesterolemia, and any lipid disorder were more frequent among participants with lower educational attainment. After adjustment for age and sex, each additional ISCED level was associated with an 11.2% lower cardiometabolic burden count. Age and education were only weakly correlated in our sample (ρ = −0.122), suggesting that this association was not simply a reflection of differences in educational attainment between older and younger participants. The finding is consistent with previous research linking education to health behaviors, use of preventive healthcare, and access to healthcare [14,16,35]. It also suggests that these socioeconomic differences remain relevant in a population characterized by subjective cognitive concerns, even in the absence of clinically significant cognitive impairment [36].

### Educational attainment and cognitive performance

Educational attainment was consistently associated with better performance across all five cognitive measures. The standardized effect sizes ranged from 0.23 to 0.43 SD per SD of educational attainment (corresponding to approximately 0.13 to 0.24 SD per additional ISCED level), which were substantially larger than the effects observed for cardiometabolic burden. This finding is consistent with our earlier analysis of this cohort [19] and the established relationship between education and cognitive functioning later in life [17,37].

An interesting aspect of the results concerns the TAP measures. The TAP T-scores are already adjusted for age, sex, and educational level, meaning that a participant’s score reflects their performance relative to individuals with a similar educational background. Despite this, education remained significantly associated with TAP performance in our sample. This residual association should be interpreted cautiously because its meaning depends on the normative adjustments applied to the scores used here.

There are several possible explanations for this finding. Participants with higher education may have been more familiar with computerized tasks, or the normative data may not fully reflect the characteristics of the Romanian population included in our study; the mean Alertness T-score of 37, more than 1 SD below the normative mean in a sample without objective cognitive impairment, would be compatible with the latter. Residual confounding by other factors associated with education may also have contributed. However, the current data do not allow us to distinguish between these explanations. The finding is nevertheless relevant when interpreting education-normed cognitive measures, particularly when they are applied to populations that may differ from the population used to establish the original normative data.

### Education as a potential moderator

Educational attainment has an important role in both parts of our findings. It was associated with cardiometabolic burden, but was also consistently related to cognitive performance, as would be expected based on the literature on cognitive reserve [38]. This raised the possibility that education might also modify the relationship between cardiometabolic burden and cognition. In other words, individuals with higher educational attainment might show less cognitive impact from a similar level of cardiometabolic burden, as has previously been reported for white matter hyperintensity burden [15].

We examined this possibility but found no clear evidence of an interaction between education and cardiometabolic burden. Four of the five interaction terms were non-significant. The only nominally significant interaction, observed for TAP Alertness, was in the opposite direction to that expected under the cognitive reserve hypothesis and should therefore be interpreted cautiously.

This finding should not be taken as evidence that cognitive reserve does not modify the effects of cardiometabolic burden. Interaction effects are generally more difficult to detect than main effects and typically require larger samples. The present study was not specifically designed or powered to investigate effect modification. In addition, the absence of a moderating effect is difficult to interpret when there was no clear association between cardiometabolic burden and cognitive performance in the first place. This question may be more appropriately examined in larger populations with a wider range of cardiometabolic burden and measurable differences in cognitive performance [39].

### Limitations

Several limitations should be considered when interpreting the findings. Cardiometabolic conditions were identified from self-reported medical history rather than direct clinical measurements. We did not have blood pressure measurements, lipid profiles or glycaemic measures available for the present analysis. As a result, undiagnosed conditions could not be identified, and the prevalence of cardiometabolic conditions may have been underestimated, particularly for hypertension, diabetes and dyslipidemia, which can remain asymptomatic. Information on disease duration, severity and treatment adequacy was also unavailable.

The eligibility criteria resulted in a relatively healthy sample compared with the general population of the same age. In particular, the BMI threshold excluded participants with obesity, despite obesity being an important cardiometabolic risk factor, while clinically significant systemic disease was excluded by design. The prevalence estimates should therefore be understood as describing this screened study population rather than the Romanian population aged 50–75 years more generally.

Another limitation concerns the construction of the cardiometabolic burden score. Each condition contributed equally to the score, regardless of its severity, duration or treatment status. Although weighted indices could potentially capture these differences more accurately, they would also introduce additional assumptions and reduce transparency and comparability with other studies. The score also counts risk conditions rather than established cardiovascular disease, so it is not equivalent to cardiometabolic multimorbidity as defined in most previous studies.

The cross-sectional design also prevents conclusions about temporal or causal relationships between cardiometabolic burden and cognitive performance. The study was conducted at a single center, and the sample was predominantly female (72.1%). Participants were also individuals who were sufficiently concerned about their cognition to volunteer for a clinical trial, which may limit the generalizability of the findings to the broader population of adults with subjective cognitive complaints. In addition, subjective cognitive complaints were used as an eligibility criterion but were not quantified, so their severity could not be examined in relation to cardiometabolic burden.

Finally, this analysis used baseline data collected as part of a clinical trial with a different primary research objective. Cardiometabolic variables were recorded as part of the participants’ medical history rather than specifically for the present research question, and the analysis was not pre-registered. These factors should be taken into account when interpreting the findings, particularly the null associations and exploratory interaction analyses.

## CONCLUSION

In a cohort of adults aged 50–75 years reporting subjective cognitive complaints and with a MoCA score above 25, cardiometabolic burden was clearly patterned by age and by educational attainment, with both gradients independent of one another. Cardiometabolic burden showed no association with objective cognitive performance once age, sex, and educational attainment were taken into account, and the estimates did not support a substantial association on the measures used in this sample. Educational attainment, by contrast, was consistently and strongly associated with cognitive performance.

The absence of an observed cross-sectional association should not be interpreted as evidence that cardiometabolic risk has no longer-term cognitive consequences. Longitudinal follow-up of similar cohorts, together with objective assessment of cardiometabolic risk and information on disease duration and severity, will be important for determining whether and when cardiometabolic burden becomes associated with measurable cognitive changes.

## Data Availability

De-identified participant data and analysis code are available from the corresponding author upon reasonable request. The study protocol is publicly available via the ISRCTN registry (ISRCTN10514154).
